# Postoperative Infection After Implant-Based Breast Reconstruction: Risk Factors and Clinical Burden in a Large Single-Center Cohort

**DOI:** 10.3390/jcm15072723

**Published:** 2026-04-03

**Authors:** Ferruccio Paganini, Beatrice Corsini, Sara Matarazzo, Elisa Bascialla, Lorenzo Fresta, Federico Lo Torto, Marco Marcasciano, Federico Tamborini, Luigi Valdatta

**Affiliations:** 1 Division of Plastic and Reconstructive Surgery, Department of Biotechnology and Life Sciences, University of Insubria, 21100 Varese, Italysmsaramatarazzo@gmail.com (S.M.);; 2 Division of Plastic Surgery, IRCCS European Institute of Oncology, 20141 Milan, Italy; 3 Division of Plastic Surgery, Surgery Department, Chieti-Pescara University, 65124 Pescara, Italy; 4 Division of Plastic Surgery, Department of Experimental and Clinical Medicine, Magna Graecia University of Catanzaro, 88100 Catanzaro, Italy

**Keywords:** implant-based breast reconstruction, postoperative infection, surgical site infection, tissue expander, breast implant, risk factors, explantation

## Abstract

**Background**: Implant-based breast reconstruction (IBBR) is the most widely used reconstructive strategy after mastectomy, but postoperative infection remains a major complication because it may require reoperation, implant explantation, and reconstructive failure. This study evaluated the incidence, determinants, and clinical burden of infection in a large single-center cohort. **Materials and Methods**: This retrospective observational study included 1537 reconstructed breasts undergoing post-mastectomy implant-based breast reconstruction. The unit of analysis was the reconstructed breast. Infection was defined clinically by erythema, pain, swelling, or secretion requiring antibiotic treatment, without requiring microbiological confirmation or formal surgical-site-infection criteria; this pragmatic definition reflects the retrospective nature of the study and should be considered when comparing results across studies. Univariate analyses were performed using chi-square, Fisher’s exact, or Mann–Whitney U tests, as appropriate. Independent predictors were assessed by multivariate binomial logistic regression. **Results**: Postoperative infection occurred in 66 of 1525 reconstructed breasts (4.3%). Among infected breasts, 54 cases (81.8%) required surgery, whereas 12 (18.2%) were managed conservatively. Implant explantation was performed in 82 of 1525 reconstructions (5.4%), and infection accounted for 39 of 74 explantations with available indication data (52.7%). In multivariate analysis, longer operative time remained independently associated with infection (OR 1.005 per minute, 95% CI 1.001–1.010; *p* = 0.010; corresponding to OR 1.38, 95% CI 1.08–1.77, per 60 min increment). Prepectoral reconstruction was also associated with a higher risk of infection compared with retropectoral reconstruction (OR 2.31, 95% CI 1.03–5.16; *p* = 0.042). Additional analyses showed that prepectoral reconstruction was more frequently associated with bilateral procedures, nipple-sparing mastectomy, and longer operative time. In unilateral reconstructions, the association between prepectoral reconstruction and infection persisted. **Conclusions**: Infection after implant-based breast reconstruction remains a clinically relevant source of morbidity and frequently requires further surgery. Longer operative time emerged as the most consistent independent factor associated with infection in the overall cohort. Prepectoral reconstruction was also associated with infection, although this finding should be interpreted cautiously in light of reconstructive context and case selection.

## 1. Introduction

Implant-based breast reconstruction (IBBR) remains the most widely adopted reconstructive option after mastectomy because of its technical versatility, lack of donor-site morbidity, and broad applicability across different clinical settings [1,2,3,4]. Despite these advantages, postoperative infection remains one of the most clinically relevant complications, given its potential to require prolonged antibiotic therapy, additional surgical procedures, implant explantation, and unplanned hospital care, with downstream consequences for adjuvant oncologic timing and patient quality of life [5,6,7,8,9,10,11,12,13].

Reported infection rates vary considerably across the literature, reflecting substantial heterogeneity in patient characteristics, reconstructive indication, device type, timing of reconstruction, adjuvant therapies, follow-up duration, and criteria used to define infectious complications [5,8,14,15,16,17,18].

Multiple patient-related, oncologic, and surgical factors have been proposed as contributors to infection risk. Smoking, diabetes, chemotherapy, radiotherapy, operative time, reconstructive plane, implant type, and the use of adjunctive materials such as acellular dermal matrix or dermal sling techniques have all been investigated, but the relative contribution of these variables remains incompletely defined and the available evidence is often inconsistent across studies [19,20,21,22,23,24,25,26,27,28]. This inconsistency is partly explained by the fact that implant-based breast reconstruction does not represent a single homogeneous procedure, but rather a broad reconstructive category encompassing tissue expanders and definitive implants, immediate and delayed procedures, different implant planes, and different adjunctive techniques.

An additional source of heterogeneity lies in perioperative management. Antibiotic prophylaxis strategies, intraoperative pocket irrigation, drain management, length of hospital stay, and postoperative antibiotic regimens vary substantially among institutions and may influence the incidence, presentation, and management of infection. As a result, comparisons between studies are often difficult, and it is not always possible to distinguish the impact of intrinsic clinical factors from that of protocol-related variability [29,30,31,32,33]. In this context, large single-center cohorts managed according to a uniform surgical and perioperative pathway may provide a more reliable framework for identifying clinically meaningful predictors of infection.

Despite the available evidence, large cohort studies combining a breast-based analytical approach, detailed surgical and perioperative variables, and a standardized institutional protocol remain limited in the literature, and the relative contribution of reconstructive and procedure-related factors within a controlled institutional setting has not been fully characterized. The present study was therefore designed to analyze postoperative infections after implant-based breast reconstruction in a large single-center cohort using the reconstructed breast as the unit of analysis, with the aims of defining the incidence of postoperative infection, identifying factors associated with its occurrence, and assessing its impact on subsequent surgical management and reconstructive outcome.

## 2. Materials and Methods

### 2.1. Study Design

A retrospective single-center observational study was conducted to evaluate postoperative infections following post-mastectomy implant-based breast reconstruction over an 11-year period at Circolo Hospital and Macchi Foundation, Varese (Italy). The analysis was performed at the level of the reconstructed breast rather than the individual patient; accordingly, each breast was treated as a separate observation. The final dataset included 1537 reconstructed breasts treated between January 2014 and May 2025. Only prosthetic reconstructions performed after mastectomy were included. Implant-based procedures performed solely for aesthetic breast augmentation or contralateral symmetrization were not considered as study units, in order to avoid potential bias related to implant placement in the presence of native glandular tissue. However, concomitant contralateral symmetrization procedures performed during the same operation were recorded as surgical variables, as they could contribute to overall operative complexity.

The cohort comprised prosthetic reconstructions performed with either tissue expanders or definitive implants and included immediate, delayed, and tertiary procedures. No smooth implants were used during the study period; all prosthetic devices included in the analysis had a textured surface. Immediate reconstructions encompassed both direct-to-implant procedures and two-stage expander-based reconstructions. For two-stage reconstructions, the two operative stages, namely tissue expander placement and subsequent expander-to-implant exchange, were considered as separate surgical events and analyzed independently when applicable. Delayed and tertiary reconstructions were also included. Reconstructions were analyzed regardless of implant plane or the use of adjunctive techniques such as acellular dermal matrix or dermal sling support.

### 2.2. Perioperative Management and Data Collection

Throughout the study period, all patients were managed according to a standardized institutional perioperative protocol. Antibiotic prophylaxis was administered 1 h before skin incision using cefazolin 2 g intravenously, or clindamycin 900 mg intravenously in cases of beta-lactam allergy. Intraoperatively, the prosthetic pocket and the implant or expander device were irrigated exclusively with sterile physiological saline solution; no antiseptic solutions were used. Pocket preparation was therefore consistently based on saline lavage rather than dry placement. Before definitive implant positioning, the surgical team routinely changed sterile gloves.

Postoperative antibiotic therapy, consisting of penicillin derivatives or fluoroquinolones in allergic patients, was started on the day of surgery and routinely continued until drain removal. Closed suction drains were used according to institutional practice and were removed once the output was below 50 mL over 24 h. Suction drainage was maintained for a maximum of 4 weeks; beyond this time point, drains were left in situ without active suction when still clinically indicated. In most cases, one or two drains were used depending on reconstructive technique and surgical extent. The consistency of this perioperative protocol across the whole cohort was considered a relevant methodological strength, as it limited management-related variability.

Clinical, oncologic, surgical, and postoperative variables were retrospectively extracted from medical records and operative reports. Demographic and baseline clinical variables included age at surgery, body mass index, menopausal status, smoking habit, and comorbidities, including diabetes, thyroid disease, dyslipidemia, hypertension, liver disease, and chronic kidney disease. Oncologic variables included chemotherapy and radiotherapy administered before reconstruction, as well as systemic treatments or radiotherapy delivered with the implant in place.

Surgical variables included type of mastectomy, prophylactic indication, axillary procedures, timing of reconstruction, implant placement at the time of mastectomy, implant plane, implant type, use of acellular dermal matrix, dermal sling techniques, concomitant flap procedures, operative time, implant volume, and bilateral contemporary surgery. Bilateral contemporary surgery was defined as implant-based reconstruction performed on both breasts during the same operation. Implant plane was categorized as retropectoral, prepectoral, or dual-plane; in this study, dual-plane referred to subpectoral implant placement with inferior prosthetic coverage provided by acellular dermal matrix or dermal sling support. Perioperative and postoperative variables included length of hospital stay, number of drains, total drain duration, duration of postoperative antibiotic therapy, and follow-up.

### 2.3. Definition of Infection and Study Outcomes

Postoperative infection was primarily defined on clinical grounds, based on the presence of erythema, pain, swelling, or wound secretion requiring antibiotic treatment. When fluid collection or wound secretion was present, microbiological cultures were obtained in order to guide antibiotic therapy. This pragmatic definition reflects the retrospective nature of the study and the available clinical documentation; it does not require microbiological confirmation or formal surgical-site-infection criteria such as those proposed by the Centers for Disease Control and Prevention. Accordingly, direct comparisons with studies using more stringent or standardized definitions should be made with caution.

The primary outcome of the study was postoperative infection rate. Secondary infection-related outcomes included the need for surgery because of infection and implant explantation due to infection. Two derived variables were created according to the timing of diagnosis: early infection, defined as infection diagnosed within 30 days after surgery, and late infection, defined as infection diagnosed more than 30 days after surgery. Because the exact date of diagnosis was not available for all infected breasts, classification as early or late infection was possible only in cases with documented timing.

### 2.4. Statistical Analysis

Descriptive statistics were used to summarize the study cohort. Normality of continuous variables was assessed using the Shapiro–Wilk test. Since most continuous variables showed non-normal distributions, they are reported as median and interquartile range, whereas categorical variables are presented as counts and percentages.

Univariate analyses were performed by comparing reconstructed breasts with and without postoperative infection. Categorical variables were analyzed using the chi-square test or Fisher’s exact test, as appropriate, while continuous variables were compared using the Mann–Whitney U test. Variables considered clinically relevant and/or associated with infection in univariate analyses were entered into a multivariate binomial logistic regression model to identify independent predictors of postoperative infection. Total drain duration and duration of postoperative antibiotic therapy were not included in the multivariate model, as both variables are more appropriately interpreted as post-event correlates of infection rather than antecedent predictors: prolonged drainage and extended antibiotic courses may reflect the clinical management of established infection rather than factors contributing to its occurrence. Results are reported as odds ratios with 95% confidence intervals.

Variables were selected for inclusion in the multivariable model on the basis of established clinical relevance from the published literature on infection after implant-based breast reconstruction, supplemented by statistical significance or borderline significance in the univariate analysis; no automated selection procedure was used. With 61 infection events included in the multivariable model and seven regression coefficients, the events-per-variable ratio was approximately 8.7, marginally below the conventional threshold of 10; results should therefore be interpreted with appropriate caution, particularly for variables with wide confidence intervals. As an additional sensitivity analysis to account for within-patient clustering in bilateral cases, a generalized estimating equations (GEE) model was fitted using an exchangeable working correlation structure, which was supported by the estimated within-patient correlation parameter (α = 0.28).

In order to better contextualize the relationship between implant plane and postoperative infection, additional exploratory analyses were performed to assess the association between implant plane and bilateral contemporary surgery, implant plane and type of mastectomy, bilateral contemporary surgery and operative time, bilateral contemporary surgery and infection, and implant plane and operative time. Associations between categorical variables were assessed using chi-square or Fisher’s exact test, as appropriate. Differences in operative time between groups were explored using independent-samples comparisons and analysis of variance.

A secondary exploratory subgroup analysis was performed among infected breasts to compare early and late infections in cases with available timing data. Statistical significance was set at *p* < 0.05. All analyses were performed using Jamovi statistical software (The Jamovi Project, version 2.6) [34].

Missing data were handled using complete-case analysis. For each variable, the denominator reflects the number of cases with available data, and both numerator and denominator are reported explicitly throughout the tables. No imputation was performed. The multivariate logistic regression model was fitted on 1472 observations with complete data across all included variables. The variables with the largest proportion of missing observations were postmenopausal status (available in 985 of 1537 cases) and timing of infection onset among infected breasts, for which 7 of 66 cases lacked sufficient documentation for classification.

## 3. Results

A total of 1537 reconstructed breasts in 728 patients were included in the analysis. Baseline demographic and oncologic characteristics are summarized in Table 1, whereas surgical, perioperative, and postoperative variables are reported in Table 2. The median age at surgery was 51 years (IQR 44–59), and the median body mass index was 23.6 kg/m^2^ (IQR 20.7–26.3). Active smoking was reported in 16.6% of cases, diabetes in 1.3%, thyroid disease in 11.9%, dyslipidemia in 8.2%, and hypertension in 19.6%. Pre-reconstruction chemotherapy had been administered in 34.0% of cases, while 20.3% received chemotherapy with the implant in place. Radiotherapy before reconstruction was recorded in 10.7% of reconstructions, and the same proportion received radiotherapy with the implant in place.

Madden mastectomy was the most common mastectomy type (58.5%), followed by nipple-sparing mastectomy (37.4%). First-stage expander reconstruction represented 44.0% of procedures, second-stage expander-to-implant reconstruction 37.7%, and direct-to-implant reconstruction 12.9%. Retropectoral placement was the most frequently used implant plane (81.6%), followed by dual-plane reconstruction (defined in this study as subpectoral implant placement with inferior coverage provided by acellular dermal matrix or dermal sling support) (11.3%) and prepectoral reconstruction (7.1%). Tissue expanders were used in 44.3% of reconstructions and definitive implants in 55.7%. Acellular dermal matrix was used in 11.2% of cases, dermal sling in 4.6%, flap-assisted reconstruction in 2.1%, and concomitant contralateral symmetrization procedures in 21.0% of cases, although implants placed solely for symmetrization were not included as study units (Table 1).

Median operative time was 145 min (IQR 108–176), median implant volume was 450 cc (IQR 370–550), median hospital stay was 4 days (IQR 3–4), median total drain duration was 12 days (IQR 8–17), and median duration of postoperative antibiotic therapy was 14 days (IQR 9–19). Median follow-up was 612 days (IQR 301–1188), corresponding to 20 months (IQR 9–38.8) (Table 2).

Postoperative infection occurred in 66 of 1525 reconstructed breasts, corresponding to an overall infection rate of 4.3% (Table 2). Among infected breasts, 54 cases (81.8%) required surgical treatment, whereas 12 cases (18.2%) were managed conservatively with antibiotic therapy. Implant explantation was performed in 82 of 1525 reconstructions (5.4%), and infection accounted for 39 of 74 explantations with available indication data (52.7%). Hematoma occurred in 3.0% of cases, seroma in 2.8%, and Baker grade III–IV capsular contracture in 5.9% (Table 2).

The univariate analysis is reported in Table 3. Thyroid disease was significantly more frequent in infected than in non-infected reconstructions (24.2% vs. 11.3%, *p* = 0.005). Acellular dermal matrix use was also significantly associated with infection (24.2% vs 10.6%, *p* < 0.001). Reconstruction timing and implant plane showed significant overall associations with infection (*p* = 0.012 and *p* = 0.002, respectively). Among continuous variables, infected breasts showed longer operative time, larger implant volume, longer drain duration, and longer postoperative antibiotic therapy. By contrast, smoking status, hypertension, pre-reconstruction chemotherapy, chemotherapy with the implant in place, axillary dissection, dermal sling use, flap-assisted reconstruction, and bilateral contemporary surgery were not significantly associated with infection (Table 3).

**Figure 1:**
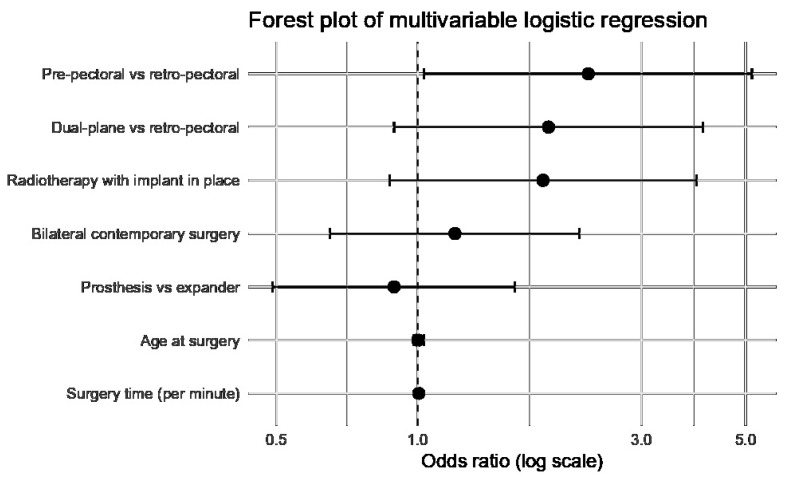
Forest plot of the multivariable logistic regression model for postoperative infection after implant-based breast reconstruction. Points represent odds ratios, and horizontal bars represent 95% confidence intervals. The vertical dashed line indicates an odds ratio of 1.

Multivariate binomial logistic regression results are reported in Table 4 and graphically summarized in Figure 1. In the adjusted model, operative time remained an independent predictor of postoperative infection (OR 1.005 per minute, 95% CI 1.001–1.010; *p* = 0.010). To facilitate clinical interpretation, this effect can also be expressed per 30 min increment (OR 1.18, 95% CI 1.04–1.33) and per 60 min increment (OR 1.38, 95% CI 1.08–1.77), indicating that each additional hour of operative time is associated with a 38% increase in the odds of postoperative infection. Prepectoral reconstruction was also independently associated with a higher risk of postoperative infection compared with retropectoral reconstruction (OR 2.31, 95% CI 1.03–5.16; *p* = 0.042). Dual-plane reconstruction showed a non-significant trend toward increased risk compared with retropectoral reconstruction (OR 1.90, 95% CI 0.89–4.06; *p* = 0.096). Radiotherapy with the implant in place also showed a non-significant trend toward increased risk (OR 1.85, 95% CI 0.87–3.93; *p* = 0.108). Age at surgery, bilateral contemporary surgery, and implant type were not independently associated with postoperative infection (Table 4, Figure 1).

As a sensitivity analysis, the multivariable model was repeated after restricting the cohort to unilateral reconstructions. In this subset, prepectoral reconstruction remained significantly associated with postoperative infection compared with retropectoral reconstruction (OR 3.12, 95% CI 1.10–8.85; *p* = 0.032), whereas operative time was no longer statistically significant (*p* = 0.188). Age at surgery, radiotherapy with the implant in place, dual-plane reconstruction, and implant type were also not significantly associated with infection in the unilateral-only model. Moreover, nipple-sparing mastectomy itself was not associated with postoperative infection in either univariate analysis (*p* = 0.662) or in an adjusted model including mastectomy type as a binary variable (OR 0.69, 95% CI 0.36–1.29; *p* = 0.241).

A further sensitivity analysis using generalized estimating equations with an exchangeable working correlation structure (α = 0.28) was performed to account for within-patient clustering in bilateral cases. Under this model, operative time remained independently associated with postoperative infection (OR 1.006, 95% CI 1.001–1.010; *p* = 0.008). The association between prepectoral reconstruction and infection was attenuated and did not reach conventional statistical significance (OR 2.34, 95% CI 0.99–5.53; *p* = 0.053), while the point estimate remained comparable to the primary analysis. All other variables showed results consistent with the primary model.

Additional exploratory analyses were performed to better contextualize the relationship between implant plane and infection risk. Bilateral contemporary surgery accounted for 505 of 1534 reconstructed breasts (32.9%), corresponding to approximately 252 patients in whom both breasts were reconstructed simultaneously and within-patient correlation is therefore structurally present at the observation level. Implant plane was significantly associated with bilateral contemporary surgery (*p* < 0.001). Bilateral procedures accounted for 29.2% of retropectoral reconstructions, 54.1% of prepectoral reconstructions, and 46.6% of dual-plane reconstructions. Implant plane was also strongly associated with type of mastectomy (*p* < 0.001). In particular, prepectoral reconstruction was predominantly performed after nipple-sparing mastectomy (81.7%), whereas retropectoral reconstruction was more commonly associated with Madden mastectomy (67.6%). However, type of mastectomy itself was not significantly associated with postoperative infection in the univariate analysis (*p* = 0.132).

Operative time significantly differed according to reconstructive plane (ANOVA *p* < 0.001). Median operative time was 142 min (IQR 105–171) for retropectoral reconstruction, 182 min (IQR 143–254) for prepectoral reconstruction, and 155 min (IQR 119–204) for dual-plane reconstruction. Bilateral contemporary surgery was associated with longer operative time, with a median of 150 min (IQR 110–204) compared with 143 min (IQR 108–170) in unilateral procedures, and a mean difference of 26.0 min (*p* < 0.001). By contrast, bilateral contemporary surgery was not significantly associated with postoperative infection in univariate analysis (*p* = 0.145) and did not remain significant in the adjusted logistic regression model.

A secondary exploratory analysis compared early infections, defined as occurring within 30 days after surgery, with late infections, defined as occurring after 30 days. Of the 66 infected breasts, timing of infection onset was documented in 59 cases (89.4%), whereas it could not be determined in 7 cases (10.6%) due to insufficient documentation. Of the 59 classifiable cases, 28 (47.5%) were classified as early infections and 31 (52.5%) as late infections. No significant differences were observed between the two groups for the evaluated categorical variables. Among continuous variables, age showed a non-significant trend toward higher values in the late infection group (Mann–Whitney *p* = 0.083), whereas no other relevant differences emerged.

## 4. Discussion

In this large single-center breast-based series, postoperative infection after implant-based breast reconstruction occurred in 4.3% of reconstructions and represented a clinically meaningful complication because it frequently required reoperation and accounted for more than half of explantations with available indication data. This incidence falls within the range generally reported in the literature for prosthetic breast reconstruction, although direct comparisons remain inherently difficult because of differences in patient selection, reconstructive timing, implant type, perioperative protocols, follow-up duration, and infection definitions [5,7,8,14,16,35,36]. In this context, the present study contributes not only incidence data, but also a more detailed assessment of the clinical burden and determinants of infection within a cohort managed under a standardized institutional protocol.

A major strength of the present study is the uniformity of perioperative patient management across the entire cohort. All patients received the same preoperative antibiotic prophylaxis, the same intraoperative pocket irrigation strategy based exclusively on saline solution, and the same postoperative policy regarding antibiotics and drain management. This aspect is particularly relevant because one of the major limitations of the available literature on infection after implant-based breast reconstruction is the marked heterogeneity in perioperative practice. Variations in antibiotic protocols, use of antiseptic irrigations, drain removal criteria, and duration of postoperative antibiotic therapy may substantially influence both infection incidence and its management, thereby complicating the interpretation of published results [29,30,33,37,38]. By reducing protocol-related variability, the present study allows a more focused evaluation of patient- and surgery-related factors.

Among the variables included in the adjusted model, operative time emerged as an independent predictor of infection. This is one of the most robust and clinically plausible findings of the present analysis. Longer operative duration is a well-established marker of procedural complexity and has repeatedly been associated with a higher risk of surgical complications across multiple surgical settings, including breast reconstruction [19]. In the specific setting of implant-based reconstruction, prolonged surgery may reflect more extensive dissection, more demanding reconstructive steps, longer prosthetic pocket exposure, or a greater degree of tissue manipulation, all of which may contribute to impaired healing or increased bacterial contamination. Although the per-minute effect size appears numerically modest, the clinical relevance becomes more apparent when expressed over longer intervals: each additional hour of operative time was associated with a 38% increase in the odds of infection (OR 1.38, 95% CI 1.08–1.77). Operative time should therefore be interpreted less as an isolated numeric variable than as a surrogate marker of overall reconstructive complexity.

The second major finding of the multivariate analysis was the association between prepectoral reconstruction and postoperative infection when compared with retropectoral placement. This result requires particularly cautious interpretation. Prepectoral reconstruction has gained increasing attention because of several recognized advantages in selected patients, including avoidance of animation deformity and potentially improved postoperative comfort, but its outcomes remain highly dependent on flap quality, soft-tissue coverage, and patient selection [39,40,41]. In the present study, prepectoral reconstruction was associated with approximately doubled odds of infection compared with retropectoral placement in the primary logistic regression model. However, this finding should not be interpreted as evidence that the prepectoral plane is intrinsically more infection-prone. Additional exploratory analyses showed that prepectoral reconstruction was strongly associated with bilateral contemporary procedures, nipple-sparing mastectomy, and longer operative time, indicating that the prepectoral group differed not only by implant plane but also by reconstructive context. In our institutional practice, prepectoral reconstruction has been preferentially used in more selected settings, including risk-reducing surgery, bilateral reconstructions, and nipple-sparing procedures, which may also involve adjunctive intraoperative steps such as perfusion assessment with indocyanine green angiography or nipple grafting—factors that were not formally modeled in the present analysis but may contribute to overall procedural complexity. The sensitivity analysis restricted to unilateral reconstructions showed that the association between prepectoral reconstruction and infection persisted (OR 3.12, 95% CI 1.10–8.85; *p* = 0.032), suggesting that the observed signal cannot be explained solely by the higher proportion of bilateral cases in that subgroup. However, when within-patient clustering was accounted for using generalized estimating equations with an exchangeable working correlation structure, the association between prepectoral reconstruction and infection was attenuated and no longer reached conventional statistical significance (OR 2.34, 95% CI 0.99–5.53; *p* = 0.053), while the point estimate remained comparable to the primary model. This pattern of attenuation is consistent with the hypothesis that a portion of the observed association reflects shared patient-level characteristics rather than an independent effect of the reconstructive plane per se. Confounding by indication therefore cannot be excluded, and reconstructive plane should be interpreted within the broader clinical and technical framework in which it is selected rather than as an isolated determinant of infectious risk. Future analyses stratifying prepectoral cases by prophylactic versus therapeutic indication would further clarify the relative contribution of plane and reconstructive context. Notably, nipple-sparing mastectomy itself was not associated with postoperative infection in either univariate analysis or after adjustment, arguing against a simple attribution of the prepectoral signal to the higher proportion of nipple-sparing procedures in that subgroup [42,43,44].

Dual-plane reconstruction showed a non-significant trend toward increased risk compared with retropectoral placement, while radiotherapy with the implant in place also showed a non-significant trend in the adjusted model. Both observations are clinically plausible. With regard to radiotherapy, the result is consistent with the well-recognized adverse biological effects of irradiation on tissue quality, wound healing, and the periprosthetic environment, even if this effect did not reach formal statistical significance in the present cohort. This may reflect limited power, residual confounding, or the fact that some of the impact of radiotherapy is mediated through reconstructive choice, flap condition, and tissue characteristics rather than acting as a completely independent variable. Similarly, the absence of an independent association for chemotherapy should not necessarily be interpreted as evidence of no effect. In this study, systemic oncologic treatments were recorded in relatively broad categories, without detailed stratification by specific regimen, timing, cumulative exposure, or combination schedules, which may have reduced the ability to detect treatment-specific effects.

By contrast, implant type (namely tissue expander versus definitive implant) did not independently influence infection risk in the final model. This suggests that, within a standardized institutional protocol, infectious risk may be driven more by procedural complexity and reconstructive context than by the simple distinction between device types. Similarly, age did not appear to independently affect infection risk in this cohort.

Another major message of the present study concerns the clinical impact of infection once established. More than 80% of infected reconstructions required surgical treatment, and infection represented the leading documented indication for explantation. This reinforces the concept that infection in prosthetic breast reconstruction should not be regarded as a minor postoperative event. Even when its absolute frequency remains within the expected range, its consequences in terms of reoperation, implant retention, reconstructive continuity, and overall treatment burden are substantial [9,10].

The exploratory comparison between early and late infections, conducted on 59 of 66 infected breasts with available timing data, did not reveal statistically significant differences in the evaluated categorical variables, while age showed only a non-significant trend toward higher values in the late infection group (Mann–Whitney *p* = 0.083). This negative result remains informative, as it argues against an obvious separation between early and late infections on the basis of the measured variables alone.

Several limitations of the present study should be acknowledged. First, the retrospective design inevitably introduces the possibility of incomplete data capture, residual confounding, and selection bias. A further limitation concerns the retrospective definition of the infection endpoint: mild infections managed entirely outside the primary center may not have been captured in institutional records, representing a potential source of underascertainment; conversely, some inflammatory presentations treated empirically with antibiotics without a confirmed infectious etiology may have met the clinical definition used in this study, introducing a risk of overcapture. The use of a clinical rather than microbiologically confirmed or CDC-criteria-based definition should therefore be considered when comparing these results with studies applying more standardized endpoints. Second, although the cohort is large for a single-center prosthetic reconstruction series, the absolute number of infection events remains limited for more complex multivariable and subgroup analyses. Third, the breast-based unit of analysis, while appropriate from a reconstructive standpoint, may introduce partial non-independence in bilateral cases, since two breasts from the same patient share systemic, oncologic, and clinical characteristics and therefore cannot be considered fully independent observations. A mixed-effects logistic sensitivity analysis with patient-level random intercept was explored but showed limited stability due to the relatively low number of infection events and the clustered data structure. A GEE analysis with exchangeable working correlation structure was therefore performed as an alternative sensitivity analysis; the estimated within-patient correlation parameter (α = 0.28) confirmed the presence of meaningful clustering. Under this model, the operative time finding was robust, whereas the prepectoral association was attenuated and did not reach conventional statistical significance, further supporting the cautious interpretive framing adopted throughout. A separate sensitivity analysis restricted to unilateral reconstructions showed persistence of the prepectoral association, indicating that this signal is not solely attributable to bilaterality. The primary logistic regression results should nonetheless be interpreted with awareness of the residual clustering structure and the events-per-variable constraint noted above. Fourth, reconstructive choices such as implant plane are strongly influenced by clinical judgment and case selection, and some degree of confounding by indication cannot be fully eliminated. Finally, the systemic treatment variables were not sufficiently granular to distinguish the potential impact of different chemotherapy, immunotherapy, or combined treatment regimens.

Despite these limitations, the present study has several strengths. It includes a large number of reconstructed breasts, applies a breast-based analytical approach, incorporates detailed surgical and perioperative variables, and reflects real-world practice under a highly standardized perioperative protocol. The supplementary contextual analyses on reconstructive plane, bilaterality, mastectomy type, and operative time improve the interpretability of the main multivariable findings by showing that variables associated with infection are also markers of broader technical and clinical complexity. Postoperative infection after implant-based breast reconstruction remains a major source of morbidity, and longer operative duration consistently emerges as a relevant associated factor in the overall cohort. The association between prepectoral reconstruction and infection should be interpreted cautiously, but its persistence in unilateral reconstructions suggests it cannot be attributed solely to the higher frequency of bilateral procedures and more likely reflects a combination of implant plane and broader reconstructive context.

## 5. Conclusions

In this large single-center breast-based series, postoperative infection after implant-based breast reconstruction occurred at a rate consistent with the published literature, but once established, it was associated with substantial clinical burden, including frequent reoperation and implant explantation. Longer operative time emerged as the most consistent independent factor associated with infection in the overall cohort, underscoring the importance of procedural complexity in determining postoperative infectious risk. Prepectoral reconstruction also showed an adjusted association with infection, and this signal persisted in a sensitivity analysis restricted to unilateral reconstructions. Nevertheless, this finding should still be interpreted with caution, because prepectoral cases were more often linked to nipple-sparing mastectomy, longer operations, and a broader pattern of case selection consistent with a more complex reconstructive context. Overall, these findings highlight the importance of careful case selection and meticulous reconstructive planning, and suggest that operative duration, as a marker of overall procedural complexity, warrants attention in the risk stratification of patients undergoing prosthetic breast reconstruction.

## Figures and Tables

**Table 1:** Baseline characteristics of the population.

Variable	Value
**Total number of patients**	728
**Number of reconstructed breasts**	1537
**Age at surgery (years)**	51 (IQR 44–59)
**Body Mass Index (kg/m^2^)**	23.6 (IQR 20.7–26.3)
**Postmenopausal status, n/N (%)**	608/985 (61.7%)
**Active smoking, n/N (%)**	240/1450 (16.6%)
**Diabetes, n/N (%)**	20/1535 (1.3%)
**Thyroid disease, n/N (%)**	182/1528 (11.9%)
**Dyslipidemia, n/N (%)**	126/1528 (8.2%)
**Hypertension, n/N (%)**	299/1528 (19.6%)
**Liver disease, n/N (%)**	8/1528 (0.5%)
**Chronic kidney disease, n/N (%)**	3/1528 (0.2%)
**Pre-implant chemotherapy, n/N (%)**	516/1519 (34.0%)
**Chemotherapy with implant in place, n/N (%)**	309/1519 (20.3%)
**Pre-implant radiotherapy, n/N (%)**	162/1517 (10.7%)
**Radiotherapy with implant in place, n/N (%)**	162/1519 (10.7%)
**Hormonal therapy before implant, n/N (%)**	378/1506 (25.1%)
**Hormonal therapy with implant, n/N (%)**	707/1501 (47.1%)
**Immunotherapy before implant, n/N (%)**	90/1513 (5.9%)
**Immunotherapy with implant, n/N (%)**	133/1512 (8.8%)
**Prophylactic mastectomy, n/N (%)**	91/1537 (5.9%)

Note: Continuous variables are reported as median (interquartile range); n: number of cases; N: total number in the group; IQR: interquartile range.

**Table 2:** Surgical characteristics, perioperative variables and complications.

Variable	Value
**Type of mastectomy**	
***Madden***	883/1509 (58.5%)
***Nipple-sparing mastectomy***	565/1509 (37.4%)
***Skin-sparing mastectomy***	47/1509 (3.1%)
***Skin-reducing mastectomy***	14/1509 (0.9%)
**Sentinel lymph node positivity, n/N (%)**	340/1399 (24.3%)
**Axillary dissection performed, n/N (%)**	445/1473 (30.2%)
**Type/timing of reconstruction, n/N (%)**	
***Direct-to-implant***	198/1537 (12.9%)
***First stage expander placement***	676/1537 (44.0%)
***Second stage expander-to-prosthesis***	579/1537 (37.7%)
***Delayed***	14/1537 (0.9%)
***Tertiary***	70/1537 (4.6%)
**Reconstruction plane, n/N (%)**	
***Retropectoral***	1252/1535 (81.6%)
***Dual-plane***	174/1535 (11.3%)
***Prepectoral***	109/1535 (7.1%)
**Implant type, n/N (%)**	
***Expander reconstruction***	680/1535 (44.3%)
***Prosthesis reconstruction***	855/1535 (55.7%)
**Implant volume (cc)**	450 (IQR 370–550)
**Flap reconstruction, n/N (%)**	32/1537 (2.1%)
**ADM use, n/N (%)**	172/1535 (11.2%)
**Dermal sling use, n/N (%)**	71/1535 (4.6%)
**Bilateral contemporary mastectomy with reconstruction, n/N (%)**	505/1534 (32.9%)
**Contralateral symmetrization, n/N (%)**	290/1383 (21.0%)
**Surgery time (min)**	145 (IQR 108–176)
**Hospital stay (days)**	4 (IQR 3–4)
**Number of drains**	1 (IQR 1–1)
**Total days with drains**	12 (IQR 8–17)
**Total days of antibiotics**	14 (IQR 9–19)
**Follow-up (days)**	612 (IQR 301–1188)
**Complications, n/N (%)**	
***Hematoma***	46/1527 (3.0%)
***Seroma***	42/1525 (2.8%)
***Infection***	66/1525 (4.3%)
***Implant explantation***	82/1525 (5.4%)
***Capsular contracture (Baker III–IV)***	75/1278 (5.9%)

Note: n: number of cases; N: total number in the group; IQR: interquartile range; ADM: acellular dermal matrix.

**Table 3:** Univariate analysis of factors associated with postoperative infection.

Variable	No Infection	Infection	*p*-Value
**Patients characteristics**			
**Age at surgery (years)**	51.0	51.5	0.789
**Patient comorbidities**			
***Active smoking, n/N (%)***	227/1382 (16.4)	11/63 (17.5)	0.829
***Diabetes, n/N (%)***	17/1457 (1.2)	3/66 (4.5)	0.052 *
***Thyroid disease, n/N (%)***	164/1456 (11.3)	16/66 (24.2)	0.005 *
***Hypertension, n/N (%)***	284/1456 (19.5)	15/66 (22.7)	0.519
**Oncologic treatment**			
***Pre-implant chemotherapy, n/N (%)***	490/1444 (33.9)	24/64 (37.5)	0.556
***Chemotherapy with implant in place, n/N (%)***	298/1444 (20.6)	10/64 (15.6)	0.330
***Radiotherapy with implant in place, n/N (%)***	150/1444 (10.4)	11/64 (17.2)	0.096 *
**Surgery characteristics**			
***Axillary dissection, n/N (%)***	426/1407 (30.3)	19/64 (29.7)	0.920
***ADM use, n/N (%)***	154/1459 (10.6)	16/66 (24.2)	<0.001
***Dermal sling use, n/N (%)***	65/1459 (4.5)	6/66 (9.1)	0.122
***Flap reconstruction, n/N (%)***	30/1459 (2.1)	2/66 (3.0)	0.589
**Bilateral contemporary mastectomy with reconstruction, n/N (%)**	471/1458 (32.3)	27/66 (40.9)	0.145
**Implant plane, n/N (%)**			0.002
***Retropectoral***	1201/1459 (82.3)	44/66 (66.7)	
***Prepectoral***	98/1459 (6.7)	11/66 (16.7)	
***Dual-plane***	160/1459 (11.0)	11/66 (16.7)	
**Type of mastectomy, n/N (%)**			0.132
***Madden***	840/1435 (58.5)	37/65 (56.9)	
***Nipple-sparing***	536/1435 (37.4)	26/65 (40.0)	
***Skin-sparing***	47/1435 (3.3)	0/65 (0)	
***Skin-reducing***	12/1435 (0.8)	2/65 (3.1)	
**Reconstruction timing, n/N (%)**			0.012
***Delayed reconstruction***	13/1459 (0.9)	1/66 (1.5)	
***Direct-to-implant***	179/1459 (12.3)	17/66 (25.8)	
***First-stage expander reconstruction***	643/1459 (44.1)	30/66 (45.5)	
***Second-stage expander reconstruction***	558/1459 (38.2)	16/66 (24.2)	
***Tertiary reconstruction***	66/1459 (4.5)	2/66 (3.0)	
**Implant volume (cc)**	450	547.5	<0.001
**Surgery time (minutes)**	144	169	<0.001
**Hospital stay (days)**	3	4	0.227
**Number of drains**	1	1	0.609
**Total days with drains (days)**	11	15	0.019
**Total days of antibiotics (days)**	14	18.5	<0.001
**Follow-up (days)**	609	701	0.315

Note: * Fisher exact test used when appropriate; n: number of cases; N: total number in the group; ADM: acellular dermal matrix; total days with drains and total days of antibiotics are reported as descriptive variables and they were not included in the multivariate model as they are more appropriately interpreted as post-event correlates of infection rather than antecedent predictors.

**Table 4:** Multivariate logistic regression analysis for predictors of postoperative infection.

Variable	Odds Ratio	95% CI	*p*-Value
**Age at surgery**	1.00	0.98–1.03	0.796
**Radiotherapy with implant**	1.85	0.87–3.93	0.108
**Reconstruction plane**			
***Prepectoral vs. retropectoral plane***	2.31	1.03–5.16	0.042
***Dual-plane vs. retropectoral***	1.90	0.89–4.06	0.096
**Bilateral contemporary mastectomy with reconstruction**	1.20	0.65–2.21	0.556
**Prosthesis vs. expander**	0.89	0.49–1.61	0.691
**Surgery time (per minute)**	1.005	1.001–1.010	0.010

Note: CI: confidence interval.

## Data Availability

The dataset supporting the findings of this study has been uploaded to the MDPI submission system and is available from the corresponding author upon reasonable and justified request. The data are not publicly available due to privacy and ethical restrictions.

## Abbreviations

The following abbreviations are used in this manuscript:

ADM	acellular dermal matrix
CI	confidence interval
GEE	generalized estimating equations
IBBR	implant-based breast reconstruction
IQR	interquartile range
OR	odds ratio
